# Einfluss der Kontaktbeschränkungen gegen SARS-CoV-2 auf die körperliche Aktivität von Beschäftigten des öffentlichen Dienstes

**DOI:** 10.1007/s40664-022-00487-5

**Published:** 2022-12-23

**Authors:** Philipp Maier, Friedrich Barsch, Oliver Morath, Oliver Krumnau, Stephan Prettin, Daniel Steinmann, Peter Deibert

**Affiliations:** 1 Institut für Bewegungs- und Arbeitsmedizin, Medizinische Fakultät, Universitätsklinikum Freiburg, Hugstetter Str. 55, 79106 Freiburg, Deutschland; 2 Betriebsärztlicher Dienst, Universitätsklinikum Freiburg, Freiburg, Deutschland

**Keywords:** COVID-19, Mitarbeitergesundheit, Gesundheitsförderung, Verhaltensänderung, Prävention, Freizeitaktivität, COVID-19, Employee Health, Health promotion, Prevention, Behavior change, Leisure activity

## Abstract

**Zielstellung:**

Erste Ergebnisse aus Aktivitätsbefragungen deuten darauf hin, dass sich die sportliche Aktivität innerhalb der Kontaktbeschränkungen zur Eindämmung des Coronavirus im Frühjahr 2020 verringert haben könnte. Die Coronavirus-Pandemie stellt im Besonderen die Beschäftigten im Gesundheitswesen vor große Herausforderungen. Daher soll untersucht werden, ob die Maßnahmen zur Pandemieeingrenzung einen Einfluss auf das Aktivitätsverhalten von Beschäftigten im öffentlichen Dienst haben.

**Methode:**

Mit einer retrospektiven Querschnittserhebung wurde das Aktivitätsverhalten in drei Einrichtungen des öffentlichen Dienstes vor und während der Maßnahmen gegen das Coronavirus im April 2020 mit einer Onlineversion des Freiburger Aktivitätsfragebogens erhoben. Mittels Wilcoxon-Tests bei verbundenen Stichproben mit einem Signifikanzniveau von *p* < 0,05 wurde das Aktivitätsverhalten auf Unterschiede vor gegenüber während den Kontaktbeschränkungen in Minuten/Woche und MET-Min/Woche untersucht.

**Ergebnisse:**

An der Befragung haben *n* = 1797 Beschäftigte (36,0 % männlich, 63,9 % weiblich und 0,1 % divers) teilgenommen. Für die Sportaktivität konnte ein relevanter (*p* < 0,05) Unterschied in den Medianen (Mdn) der Aktivitäten pro Woche vor (Mdn = 180 min) und während (Mdn = 120 min) der geltenden Kontaktbeschränkungen gemessen werden. Auch der Energieverbrauch durch Aktivität sank innerhalb der Kontaktbeschränkungen.

**Schlussfolgerung:**

Die Maßnahmen zur Pandemieeingrenzung haben zu einer Reduzierung des Aktivitätsverhaltens geführt. Insbesondere treiben weniger Beschäftigte Sport. Dies könnte in Zusammenhang mit der Schließung von Fitnessstudios stehen, da diese Aktivitäten besonders reduziert wurden. Die reduzierte Aktivität kann zu ungünstigen individuellen Risikoprofilen führen, die es in der Folge zu kompensieren gilt.

## Abstract (en)

**Objective:**

Preliminary results from activity surveys conducted in spring 2020 suggest that athletic activity may have decreased within the contact restrictions against the spread of coronavirus. The coronavirus pandemic poses many challenges to the workforce in the healthcare system. Therefore, this study investigated whether the measures to limit the pandemic have an influence on the activity behavior of employees in the public sector.

**Method:**

A retrospective cross-sectional survey was conducted to collate the activity behavior among employees of three institutions in the public sector before and during the measures against the coronavirus in April 2020. An online version of the Freiburg Activity Questionnaire was used. Using Wilcoxon tests on connected samples with a significance level of *p* < 0.05, the activity behavior was examined for differences before compared to during the contact restrictions in min/week and MET-min./week.

**Results:**

A total of 1797 public sector employees in Freiburg (36.0% male, 63.9% female, and 0.1% diverse) participated in the survey. For sports activity, a relevant difference (*p* < 0.05) was measured in the medians (Mdn) of activities per week before (Mdn = 180 min) and during (Mdn = 120 min) the relevant contact restrictions. Similarly, for energy expenditure through exercise, the median value within the contact restrictions decreased from Mdn = 1022 MET-min/week to Mdn = 750 MET-min./week.

**Conclusion:**

Measures to limit the spread of the coronavirus have led to a reduction in activity levels among public sector employees. In particular, fewer employees engaged in sports. This could be related to the closure of fitness studios as these activities were particularly reduced. Decreased physical activity can lead to unfavorable individual risk profiles, which must be compensated for in the future.

## Hintergrund

Im März 2020 rief die Weltgesundheitsorganisation (WHO) den Ausbruch des neuartigen Coronavirus (SARS-CoV-2) zu einer globalen Pandemie aus. Neben entsprechenden Hygienemaßnahmen und Abstandsregelungen waren Quarantäne sowie zeitweise Schließungen verschiedener öffentlicher Einrichtungen seitens der Länderregierungen verordnet worden, um die Infektionszahlen zu senken. Von der Landesregierung Baden-Württemberg wurde mit Inkrafttreten zum 16.03.2020 eine entsprechende Verordnung kommuniziert [8]. Diese beinhaltet unter § 1a Punkt 6 auch weitreichende Einschränkungen zur Freizeitgestaltung. Diese Einschränkungen lassen auch Veränderungen insbesondere im Gesundheitsverhalten erwarten, da die Sportmöglichkeiten durch Schließungen von Fitnessstudios und Einstellung des Vereinssportes ab diesem Zeitpunkt begrenzt waren. Dabei ist zu berücksichtigen, dass nach Angaben des Arbeitgeberverbandes deutscher Fitness- und Gesundheitsanlagen ca. 11,7 Mio. Deutsche Mitglieder eines Fitnessstudios sind [22]. Innerhalb des Deutschen Olympischen Sportbundes sind nach eigenen Angaben über 27,5 Mio. Deutsche in Individual- und Mannschaftssportarten aktiv.

Der Begriff „körperliche Aktivität“ definiert jede Form körperlicher Bewegung, welche durch die Skelettmuskulatur produziert wird und den Energieverbrauch über den Grundumsatz anhebt [19]. Körperliche Aktivität vereint demnach sowohl das organisierte Sporttreiben in Vereinen wie auch das gesundheitsbewusste Bewegen (Radfahren, Spazierengehen etc.) in der Freizeit. Regelmäßige körperliche Aktivität führt zu mannigfaltigen positiven Effekten auf die Gesundheit des Menschen. Unter anderem zeigen Untersuchungen einen positiven Einfluss von körperlicher Aktivität auf die Vermeidung von Herz-Kreislauf-Erkrankungen, Stoffwechselkrankheiten sowie Erkrankungen des Bewegungsapparates. Menschen, die körperlich aktiv sind, sind seltener übergewichtig, haben ein geringeres Risiko für Bluthochdruck oder Diabetes und zeigen ein günstigeres Lipidprofil [4]. Zudem steigert regelmäßige körperliche Aktivität die kardiorespiratorische Fitness und führt damit zu einer generellen Zunahme der körperlichen Leistungsfähigkeit, einer verbesserten Lebensqualität sowie zu einer höheren Lebenserwartung [3, 9, 14, 18]. Darüber hinaus besteht eine Evidenz für die Wirksamkeit körperlicher Aktivität bezüglich der Stabilisierung bei psychoaffektiven Erkrankungen sowie der Resilienz und stellt ein wirksames Mittel gegen wahrgenommenen Stress dar [12]. Die WHO empfiehlt regelmäßige körperliche Aktivität bei moderater Intensität mit einer Dauer von mindestens 150 min/Woche oder bei höherer Intensität mit einer Dauer von 75 min/Woche. Dabei können 10-minütige Einheiten addiert werden. Zusätzlich wird ein Krafttraining für alle großen Muskelgruppen an 2 Tagen in der Woche empfohlen [23]. Laut einer Erhebung aus dem Jahr 2015 erreichen in der Bundesrepublik Deutschland knapp 43 % der Frauen und 48 % der Männer die empfohlenen 150 min körperliche Aktivität in der Woche. Wird das Krafttraining hinzugenommen, verringert sich die Anzahl für beide Geschlechter auf ca. 20 % der Befragten [17]. Bereits eine Inaktivität über wenige Wochen vermag bei anhaltend verringerter Fitness auch bei jungen Gesunden eine deutliche Einbuße der körperlichen Leistungsfähigkeit zu bewirken [15, 26]. Eine Verschlechterung in jungem oder mittlerem Lebensalter kann bereits nach wenigen Jahren eine reduzierte metabolische Fitness bzw. erhöhte Letalität bedingen [13, 20, 21].

Die Pandemie führt insbesondere im Gesundheitswesen zu außerordentlichen Belastungen in unbekanntem Ausmaß. So ist beispielsweise das Ansteckungs- und damit Erkrankungsrisiko für das medizinische Personal höher. Aktuelle Studien zeigen zudem, dass die Beschäftigten von Kliniken über ein erhöhtes Maß an Stresserleben, depressiven sowie ängstliche Symptomen berichten [2, 16]. Die Prävention und Reduktion psychischer Belastung von Gesundheitsfachkräften ist aus diesem Grund von zentraler Bedeutung und spielt auch bei der Aufrechterhaltung der Funktionsfähigkeit des Gesundheitssystems eine wichtige Rolle [16]. Der Einfluss der Einschränkungen auf die Freizeitgestaltung (z. B. Quarantäne, Ausgangssperren) im Rahmen einer Pandemie auf sowohl die körperliche wie auch psychomentale Belastbarkeit ist unklar. Denkbar ist eine starke Abnahme der körperlichen Aktivität durch direkte Einschränkung der Bewegungsfreiheit der Personen wie auch indirekte Beeinflussung durch Wegfall des Angebotes (z. B. Schließung von Fitnessstudios, Schwimmbädern etc.) oder Verbot durch Kontaktbeschränkungen (z. B. Mannschaftssportarten). Anders könnte es allerdings bei Beschäftigten im Homeoffice oder mobilen Arbeiten sein. Einer Metanalyse zur Folge kann das Arbeiten zu Hause das Autonomieempfinden von Beschäftigten fördern und das Risiko von Work-Family-Konflikten reduzieren [25]. Zudem legen Untersuchungen während der Corona-Pandemie nahe, dass das mobile Arbeiten die arbeitsbezogenen Stressoren und Ressourcen und damit die generelle Arbeitsfähigkeit („work ability“) beeinflussen könnte [1]. Dies wiederum kann einen positiven Effekt auf das metabolische Risiko, die Lebensqualität und die Lebenserwartung haben. Die gewonnene Zeit könnte in sportliche Aktivitäten investiert werden und eine etwaige Förderung von sportlichen Aktivitäten darstellen.

In den USA kam es durch entsprechende Schutzmaßnahmen gegen die Pandemie zu einer Veränderung des Gesundheitsverhaltens, was u. a. eine leichte aber stetige Gewichtszunahme bei einer longitudinalen Kohortenstudie zur Folge hatte [10]. Erste Ergebnisse aus Aktivitätsbefragungen im Frühjahr 2020 aus Deutschland und weiteren Ländern deuten zudem darauf hin, dass sich die sportliche Aktivität in der Bevölkerung durch die Corona-Pandemie verringert haben könnte [11, 24]. Im März 2020 sind von Fitness-Trackern bereits erste Aktivitätszahlen veröffentlicht worden, die auffallend verringerte Werte in der Anzahl der Schritte pro Tag zeigen [5]. Daraus ergeben sich bereits proaktive Empfehlungen für Aktivitäten, die auch zu Hause und während einer Quarantäne durchgeführt werden können [7]. Welche Auswirkungen die Corona-Pandemie in Kombination mit einschränkenden Maßnahmen auf das Aktivitätsverhalten von Beschäftigten im öffentlichen Dienst hat, ist Gegenstand dieser Studie. Dabei wird erwartet, dass sich das Aktivitätsverhalten der Beschäftigten im Vergleich zwischen vor und während den einschränkenden Maßnahmen messbar verändert.

## Methode

### Stichprobe

Zur Studie eingeladen waren alle Beschäftigten über 18 Jahre aus drei Freiburger Einrichtungen des öffentlichen Dienstes: des Universitätsklinikums Freiburg, des Landratsamts Breisgau-Hochschwarzwald sowie des Regierungspräsidiums Freiburg. Rekrutiert wurden sie über einen Aufruf zur Teilnahme im Intranet der jeweiligen Einrichtung.

### Studiendesign

Mittels Online-Fragebogen wurde eine retrospektive Querschnittserhebung durchgeführt, welche das Aktivitätsverhalten vor und während der Maßnahmen gegen das Coronavirus erfassen sollte. Für eine einheitliche Aufteilung der beiden Zeiträume wurde die Grenze am Tag der von der Landesregierung Baden-Württemberg verordneten Maßnahmen zur Einschränkung des öffentlichen Lebens (Kontaktbeschränkungen, Schließung privater u. öffentlicher Sportanlagen) gewählt. In Baden-Württemberg war der Beginn dieser Maßnahmen der 16. März 2020. Die Befragung wurde ab April 2020 über einen Zeitraum von 14 Tagen durchgeführt.

### Fragebogen

Das Aktivitätsverhalten wurde anhand des Freiburger Aktivitätsfragebogen [6] ermittelt. Dieser teilt die Aktivitäten in Basisaktivitäten (Einkaufen oder zur Arbeit gehen), Freizeitaktivitäten (z. B. Spazierengehen) und Sportaktivitäten (z. B. Joggen). Dabei sollten die Probanden die jeweiligen Aktivitäten in Minuten pro Woche angeben. Dazu zählte jede Form körperlicher Bewegung, die mindestens 10 min am Stück durchgeführt wurde. Zusätzlich wurden demografische Angaben über Geschlecht, Altersgruppe sowie Berufsgruppe am Klinikum erfragt. Der Freiburger Aktivitätsfragebogen wurde in eine Onlineversion übertragen und entsprechend der Fragestellung modifiziert. Verwaltet wurden die Daten durch eine browserbasierte Datensoftware (REDCap®, Vanderbilt University, Nashville, TN, USA, https://projectredcap.org/), auf welcher der Fragebogen hinterlegt und über einen Link abrufbar war.

### Datenanalyse

Die Angaben zu einzelnen Aktivitäten wurden in Basis‑, Freizeit- und Sportaktivitäten in Minuten pro Woche für die Zeiträume vor und während den Beschränkungen zusammengefasst. Es wurden die Mittelwerte, Standardabweichungen sowie Mediane zwischen den einzelnen Aktivitäten verglichen. Neben der Dauer wurde der Energieverbrauch der jeweiligen Aktivitäten mit Hilfe des metabolischen Äquivalents (MET) geschätzt und in MET-Minuten pro Woche (MET-Min/Woche) analysiert. Daraus wurde eine kategorische Einteilung in Abhängigkeit des Energieverbrauchs in Inaktive (< 600 MET-Min/Woche), Minimalisten (600–1200 MET-Min/Woche) und Aktive (> 1200 MET-Min/Woche) gebildet. Zur Datenanalyse wurde SPSS 25 (IBM Corp., Armonk, NY, USA) oder höher verwendet. Die deskriptive Auswertung und Charakterisierung der Fälle und Vergleichsperson bezüglich Alter, Geschlecht und Berufsgruppe erfolgte durch Chi-Quadrat-Test bzw. Mann-Whitney-Test. Die Verteilung der Ergebnisse des Fragebogens wurde variablenweise auf Normalverteilung mit dem Kolmogorov-Smirnov-Test analysiert. Im Falle einer Normalverteilung erfolgt der Vergleich der Aktivitäten durch Verwendung des T‑Tests für abhängige Stichproben, bei nicht normalverteilten Variablen erfolgt die Analyse mit dem Wilcoxon-Vorzeichen-Rang-Test. Als signifikant werden Unterschiede mit einer Irrtumswahrscheinlichkeit < 5 % angesehen. Zum Ausschluss eines Non-Response-Bias wurden die frühsten eingegangenen 10 % der Antworten mit den spätesten 10 % der eingegangenen Antworten verglichen. Es wird davon ausgegangen, dass ein nicht signifikanter Unterschied der beiden Gruppen einen entsprechenden Bias ausschließt.

## Ergebnisse

An der Befragung haben *n* *=* 1797 Beschäftigte des öffentlichen Dienstes (Universitätsklinikum Freiburg *n* = 731, Regierungspräsidium Freiburg *n* = 561, Landratsamt Freiburg *n* = 505) teilgenommen. Tab. 1 zeigt die Verteilung für die Geschlechts- und Altersgruppen.

**Table 1:** 

		*N* (%)
*Gesamt*	**–**	1797
*Geschlecht*	Weiblich	1152 (63,9)
	Männlich	644 (36,0)
	Divers	1 (0,1)
*Institution*	Uniklinik	731 (40,7)
	Regierungspräsidium	561 (31,2)
	Landratsamt	505 (28,1)
*Altersgruppe*	18–29 Jahre	301 (16,8)
	30–44 Jahre	574 (31,9)
	45–64 Jahre	910 (50,6)
	+65 Jahre	12 (0,7)

### Veränderung der Aktivität

Das Aktivitätsverhalten wurde in Minuten pro Woche erfasst. Für die Basis, Freizeit- und Sportaktivität zeigt Tab. 2 die Lage- und Streuungsmaße für die beiden Zeiträume. Bei der Sportaktivität waren die wöchentlich durchgeführten Aktivitätszeiten in Minuten pro Woche während den Kontaktbeschränkungen signifikant geringer (*Mdn* = 120,0 min) als vor den Beschränkungen (*Mdn* *=* 180,0 min), *z* = −11,304; *p* < 0,05. Die Effektstärke wies mit r = −0,266 einen schwach mittleren Effekt auf. Die Freizeitaktivität hat während der Beschränkungen (Mdn = 100 min) im Gegensatz zu davor (Mdn = 63 min) signifikant zugenommen (z = 14,639). Der Unterschied in der Freizeitaktivität wies einen mittleren Effekt von r = 0,345 auf. Werte für die Basisaktivität sind Tab. 2 zu entnehmen und sind für beide Zeiträume auf nahezu identischem Niveau geblieben.

**Table 2:** 

	Vor			Während		
	M (SD)	Median	Min–Max	M (SD)	Median	Min–Max
Basisaktivität	170,4 (192,1)	122	0–2501	150,7 (173,8)	101*	0–2008
Freizeitaktivität	113,7 (143,5)	63	0–1262	141,8 (166,9)	100*	0–1263
Sportaktivität	234,0 (212,3)	180	0–1200	193,5 (206,8)	120*	0–1120

Angaben in Minuten/Woche

* *p* < 0,05

Auch für den Energieverbrauch durch sportliche Aktivität sank der Wert im Median innerhalb der Kontaktbeschränkungen von *Mdn* = 1014 MET-Min/Woche auf *Mdn* = 720 MET-Min/Woche. Bei der Freizeitaktivität ist der Energieverbrauch von *Mdn* *=* 218 MET-Min/Woche auf *Mdn* = 350 MET-Min/Woche leicht angestiegen. Tab. 3 zeigt die Ergebnisse der Veränderungen des Energieverbrauchs in MET-Min/Woche für alle drei Aktivitäten.

**Table 3:** 

	Vor			Während		
	M (SD)	Median	Min–Max	M (SD)	Median	Min–Max
Basisaktivität	672,8 (767,4)	480	0–10.000	593,2(693,2)	400*	0–8000
Freizeitaktivität	398,5 (504,1)	218	0–4418	499,3 (587,7)	350*	0–4422
Sportaktivität	1342,2 (1319,2)	1014	0–8400	1163,0 (1320,6)	720*	0–8772

Angaben in MET-Minuten/Woche

* *p* < 0,05

Zudem zeigt Tab. 4 die Einteilung in drei verschiedene Aktivitätsgruppen in Abhängigkeit des Energieverbrauchs. Mehr als die Hälfte der Befragten wiesen einen geringen oder keinen Energieverbrauch durch sportliche Aktivität auf. Der Anteil derjenigen, die keinen oder nur einen geringen Energieverbrauch durch sportliche Aktivität aufweisen, hat im Vergleich zwischen vor und während der Kontaktbeschränkungen auch am stärksten zugenommen.

**Table 4:** 

Aktivitätsgruppen	Vor (%)	Während (%)
Inaktive (< 600 MET-Min/Woche)	35,2	43,8
Minimalisten (601–1200 MET-Min/Woche)	21,3	19,3
Aktive (> 1200 MET-Min/Woche)	43,5	36,9

### Aktivität nach Institution und WHO-Empfehlung

**Figure 1:**
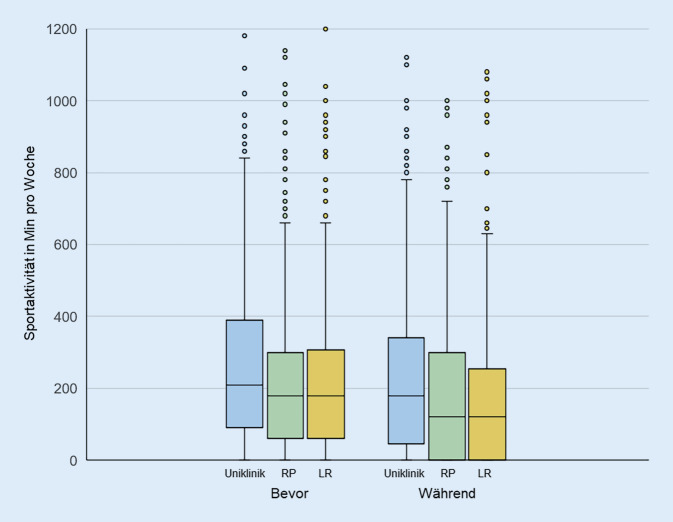

Anhand der empfohlenen Umfänge für moderate körperliche Aktivität zeigt sich, dass vor den Kontaktbeschränkungen 58 % der Befragten die Aktivitätsempfehlungen der WHO erreichten. Dieser Wert sank während der Maßnahmen auf 46,2 %. Im Gegensatz dazu stieg der Anteil der Inaktiven von 16,6 % auf 26,3 % an. Diese Veränderungen im Aktivitätsverhalten zeigt sich über alle drei Institutionen hinweg. Der Anteil derer, die einen geringen Umfang an Aktivität aufweisen, blieb innerhalb der Kontaktbeschränkungen nahezu auf demselben Niveau wie vor den Beschränkungen. Abb. 1 zeigt die Veränderungen der Sportaktivität für die drei Institutionen. Entsprechend der unterschiedlichen individuellen Lebensstile wiesen die Umfänge sportlicher Aktivität eine sehr große Streuung auf. Es ist außerdem festzuhalten, dass das Aktivitätsniveau der Befragten aus dem Uniklinikum leicht höher war als das der beiden Behörden.

## Diskussion

Mit unserer Befragung wurde untersucht, welchen Einfluss die Kontaktbeschränkungen in der Bundesrepublik Deutschland im März und April 2020 auf das Aktivitätsverhalten von Beschäftigten des öffentlichen Dienstes haben. Unsere Ergebnisse legen nahe, dass sich das Gesamtvolumen an Aktivität während der Kontaktbeschränkungen verringert hat. Insbesondere diejenige Form von Aktivität, welche zum Selbstzweck durchgeführt und damit als Sportaktivität definiert wird, hat sich in der Zeit der Kontaktbeschränkung verringert [19]. Entsprechend hat die Zahl der Beschäftigten ohne körperliche Betätigung dramatisch zugenommen. Auch bei ehemals sportlich aktiven Personen konnte der Einbruch des Sporttreibens nicht durch eine Erhöhung der Freizeitaktivitäten kompensiert werden. Unsere Ergebnisse sind mit ähnlichen Untersuchungen vergleichbar, die innerhalb des ersten Lockdowns ebenfalls Aktivitätsbefragungen in der Allgemeinbevölkerung durchgeführt haben und von einem Rückgang der Sportaktivität zwischen 30 und 40 % berichten [11, 24].

Generell ist festzuhalten, dass die befragten Beschäftigten hohe Differenzen in ihrem Aktivitätsverhalten aufweisen. Im Vergleich zu den regelmäßig durchgeführten Aktivitätsbefragungen in Deutschland erreichen mit 58 % der Befragten ähnlich viele die Aktivitätsempfehlungen vor den Kontaktbeschränkungen [4, 19]. Mehr als die Hälfte der befragten Beschäftigten sind während der Kontaktbeschränkungen allerdings unzureichend aktiv. Dabei ist insbesondere zu berücksichtigen, dass viele Beschäftigte angegeben haben, in Fitnessstudios aktiv zu sein, welche aufgrund der geltenden Verordnung in Baden-Württemberg geschlossen waren [8]. Es ist zu erwarten, dass dieser Einbruch des Aktivitätsverhaltens auch für andere Bevölkerungsgruppen zutrifft, wenn berücksichtigt wird, dass mehr als 11 Mio. Menschen in Deutschland Mitglied in einem Fitnessstudio sind [22].

Bei den Freizeitaktivitäten wurden alle körperlichen Bewegungen zusammengefasst, die nicht unter sportlicher Aktivität im engeren Sinn zu verstehen sind. Dazu gehört das Spazierengehen oder die Gartenarbeit. Diese Aktivitäten haben innerhalb der Kontaktbeschränkungen leicht zugenommen. Allerdings wird aus der Analyse nicht erkennbar, ob hier vielmehr ein witterungsbedingter Einfluss vorliegt als ein Einfluss der Kontaktbeschränkungen. Eine Kompensation fehlender sportlicher Aktivität durch eine Erhöhung der Freizeitaktivität hat offensichtlich nicht stattgefunden. Der leichte Rückgang bei den Aktivitäten zum Einkaufen oder Arbeitsweg könnte sich durch eine Veränderung der Arbeitssituation ergeben haben. Interessanterweise gab es hier keinen Unterschied zwischen den Beschäftigten der einzelnen Institutionen, was aufgrund der unterschiedlichen Tätigkeitsfelder zu erwarten wäre.

Im ähnlichen Maße verhält es sich auch mit dem Gesamtenergieverbrauch durch körperliche Aktivität, welcher eine Summierung der Produkte aus Leistung bzw. Intensität und Dauer der Aktivitäten darstellt. Es ist nicht davon auszugehen, dass die Reduzierung des Umfangs an sportlichen Aktivitäten durch kürzere und intensivere Einheiten kompensiert wurde. Somit ergeben sich Anzeichen dafür, dass die von der Regierung verhängten Maßnahmen zur Einschränkung des öffentlichen Lebens aufgrund des verringerten Aktivitätsverhaltens zu einem erhöhten Erkrankungsrisiko führen könnten, sofern das Aktivitätsverhalten nicht angepasst wird. Vorausgehende Untersuchungen haben gezeigt, dass sich bei langanhaltendem Mangel an Aktivität das Risiko für kardiovaskuläre Erkrankungen erhöht und auch die wahrgenommene psychische Belastung durch fehlende Bewegung ansteigen kann [4, 14]. Dabei ist es zur Förderung der Mitarbeitergesundheit wichtig, insbesondere auf diejenigen Beschäftigten zu fokussieren, die innerhalb der Kontaktbeschränkungen überhaupt nicht körperlich aktiv sind. Auch Aktivitäten unterhalb des eigentlich empfohlenen Umfangs bringen verschiedene gesundheitsfördernde Effekte mit sich [23]. Gerade unter der Berücksichtigung der aktuellen Herausforderungen der Corona-Pandemie könnte die Integration von regelmäßiger körperlicher Aktivität auf ganz verschiedenen Ebenen zu einer Verbesserung der Lebensqualität führen und den gesundheitlichen Folgen für die Beschäftigten unterschiedlich entgegenwirken.

Als weitere Auffälligkeit ließ sich bezüglich der Art der körperlichen Aktivität eine Zunahme des Anteils von Ausdaueraktivitäten feststellen. Dies könnte mit Schließungen von Fitnessstudios im Zusammenhang stehen. Denkbar ist aber auch eine witterungsbedingte Verlagerung der Aktivität nach draußen. Zumindest könnte sich der Zuwachs an getätigter Freizeitaktivität durch entsprechende Wettereinflüsse erklären lassen. Möglicherweise führte auch eine etwaige einfachere Umsetzbarkeit von Ausdauertraining im Vergleich zu anderen Aktivitäten zu der Veränderung, da Ausdauersportarten weniger Equipment benötigen und ein entsprechender Mindestabstand besser eingehalten werden könnte.

Durch die Nutzung eines Online-Fragebogens ist es uns gelungen, ein Kollektiv von 1797 Beschäftigten aus drei Institutionen für die Befragung zu gewinnen. Hervorzuheben ist, dass der eingesetzte Freiburger-Aktivitäts-Fragebogen eine Differenzierung der ausgeführten Aktivität ermöglicht [6]. Hierdurch konnten Aktivitätsunterschiede in verschiedenen Alltagssituationen erfragt werden. Außerdem lässt die Fragebogenerhebung auch die subjektive Wahrnehmung der Befragten zu und führt in Kombination mit einem großen Kollektiv zu einem Überblick über das Aktivitätsverhalten. Ob allerdings ein direkter Einfluss der Pandemie auf die jeweiligen Aktivitäten vorliegt, lässt sich mit unserer gewählten Form der Befragung nicht zweifelsfrei belegen. Allerdings kam es zu Verschiebungen in der Art des Sportreibens. Dies ist unter anderem durch die Methodik einer retrospektiven Fragebogenerhebung begründet. Intensitäten und die tatsächlich geleistete Bewegung könnten durch andere Tools (z. B. Wearables) erfasst werden, welche allerdings einen erhöhten Mehraufwand in der Durchführung und der Analyse erfordern und dadurch automatisch ein kleineres Kollektiv an Befragten bedingen.

Ziel war es, eine Online-Befragung bei niedriger Hemmschwelle durchzuführen, die während der Arbeitszeit durchgeführt werden kann. Daher wurde der Fragebogen bewusst kurzgehalten. Die Integration weiterer Parameter in die Befragung hätte gegebenenfalls ermöglicht, die beobachteten Aktivitätsunterschiede noch feiner zu analysieren. Insbesondere die wahrgenommenen psychischen Arbeitsbelastungen der Klinikbeschäftigten hätten noch spezifischer durch entsprechende Items oder Messungen erfasst werden können. Dies hätte jedoch die erforderliche Bearbeitungszeit deutlich erhöht, was gerade bei der Zielgruppe der Pflegenden oder ärztlichen Beschäftigten weniger Rückmeldung ergeben hätte. Zudem haben wir nicht erfasst, ob weitere einschneidende Maßnahmen, wie beispielsweise eine Kinderbetreuung durch Schulschließungen o. Ä. zu einer geringeren Freizeit und damit einem geringeren Aktivitätsverhalten geführt haben.

Innerhalb dieser Untersuchung ist mit den Beschäftigten eines Universitätsklinikums eine von der Corona-Pandemie speziell betroffene Population mituntersucht worden. Nicht nur, dass auch für Klinikpersonal dieselben Einschränkungen in der Freizeitgestaltung wie für den Rest der Bevölkerung gelten. Hinzu kommt eine erhöhte Arbeits- und Stressbelastung des Klinikpersonals im Rahmen der aktuellen Pandemie [16]. Die Auswirkungen der Maßnahmen gegen die weitere Ausbreitung des Coronavirus für Klinikbeschäftigte sind daher aus gesundheitsfördernder Sicht von besonderem Interesse. Generell scheint der Bedarf an aktivitätsförderlichen Angeboten für Klinikbeschäftigte wie auch für Beschäftigte weiterer Institutionen des öffentlichen Dienstes vorhanden zu sein. Aus den erhobenen Daten ergeben sich für uns mehrere Empfehlungen an die Beschäftigten der jeweiligen Institutionen.

Die Beschäftigten sollten über die Bedeutung von regelmäßiger körperlicher Aktivität auch und gerade während geltender Kontaktbeschränkungen aufgeklärt werden. Realisierbare Aktivitäten zur Verbesserung der Ausdauer sollten an der frischen Luft durchgeführt werden und bringen schon bei moderater Intensität und auch kurzen Zeitintervallen positive Effekte [7]. Außerdem können digitale Bewegungsangebote zur Kräftigung oder zur Verbesserung der Balance mit dem eigenen Körpergewicht oder entsprechenden Hilfsmitteln von zu Hause aus durchgeführt werden. Unabhängig der derzeitigen pandemischen Lage sollte aus Sicht des Arbeitgebers das Aktivitätsverhalten der jeweiligen Berufsgruppen evaluiert und besonders die inaktiven Beschäftigten zu mehr Aktivität und Bewegung im Alltag motiviert werden. Dies dient der Gesunderhaltung und auch langfristig der Optimierung des individuellen gesundheitlichen Risikos.

## Fazit für die Praxis

- Insbesondere im Falle von Kontaktbeschränkungen sollten Beschäftigte auf die Bedeutung und Wichtigkeit sportlicher Aktivität aufmerksam gemacht werden und entsprechend der jeweiligen behördlichen Vorgaben Aktivität und Bewegung an der frischen Luft in entsprechendem Umfang und mit moderater Intensität durchführen.
- Außerdem können digitale Fitnessangebote sowie die häusliche Umgebung (Treppensteigen) genutzt werden, um den Wegfall entsprechender Angebote in Fitnessstudios zu kompensieren.
- Darüber hinaus sind unter der Berücksichtigung einer ganzheitlichen Aktivitätsempfehlung auch Balance- und Gleichgewichtsübungen zu empfehlen und sehr leicht zu Hause durchzuführen.
- Entscheidend ist die Kenntnis über den Nutzen von körperlicher Aktivität. Daraus resultiert eine verbesserte Gesundheitskompetenz.
